# LncRNA XIST promotes extracellular matrix synthesis, proliferation and migration by targeting miR-29b-3p/COL1A1 in human skin fibroblasts after thermal injury

**DOI:** 10.1186/s40659-019-0260-5

**Published:** 2019-09-20

**Authors:** Wei Cao, Youping Feng

**Affiliations:** Department of Plastic Surgery, Tongji Hospital Affiliated to Tongji Medical College, Huazhong University of Science and Technology, No. 1095 Jiefang Rd, Wuhan, 430030 China

**Keywords:** Wound healing, Thermal injury, XIST, miR-29b-3p, COL1A1

## Abstract

**Background:**

Long noncoding RNAs (lncRNAs) have been reported to be associated with dermis process during burn wound healing. This study aimed to investigate the role of lncRNA X-inactive specific transcript (XIST) in human skin fibroblasts (HSF) and extracellular matrix (ECM) as well as the regulatory network of XIST/microRNA-29b-3p (miR-29b-3p)/collagen 1 alpha 1 (COL1A1).

**Methods:**

The wound samples were collected from 25 patients with deep partial thickness burn at day 5 after burn. The thermal injured model was established using HSF cells. The expressions of XIST, miR-29b-3p and COL1A1 were measured by quantitative real-time polymerase chain reaction and western blot. ECM synthesis, cell proliferation and migration were detected by western blot, cell counting kit-8 and trans-well assays, respectively. The interaction between miR-29b-3p and XIST or COL1A1 was explored by bioinformatics analysis and luciferase reporter assay.

**Results:**

The expressions of XIST and COL1A1 were enhanced but miR-29b-3p expression was decreased after thermal injury. XIST overexpression promoted ECM synthesis, cell proliferation and migration in thermal injured HSF cells. However, XIST knockdown played an opposite effect. miR-29b-3p overexpression inhibited ECM synthesis, cell proliferation and migration, which was reversed by XIST. COL1A1 silence suppressed ECM synthesis, cell proliferation and migration by miR-29b-3p targeting. Moreover, COL1A1 up-regulation weakened the effect of XIST silence on ECM synthesis and HSF cell function.

**Conclusion:**

XIST promoted ECM synthesis, cell proliferation and migration by sponging miR-29b-3p and targeting COL1A1 in HSF cells after thermal injury, indicating the promoting role of XIST in wound healing.

## Introduction

The therapy of deep burn wound is challenged because of the scar formation and poor function after wound healing. The dermis plays an essential role in wound repair through maintaining human skin fibroblast function and extracellular matrix (ECM) synthesis to improve scar quality [1]. On injury, fibroblasts would be activated to restore tissue integrity. ECM metabolism is the key event during wound healing, which indicates the repair quality and outcomes [2]. However, the molecular mechanism in wound healing has not been fully reported.

Long noncoding RNAs (lncRNAs) exert their functions by acting as sponges of microRNAs (miRNAs) or competing endogenous RNAs (ceRNAs) for miRNAs to derepress mRNAs [3]. Moreover, lncRNAs have been suggested to play vital roles in wound healing after thermal injury. For example, lncRNA low expression in tumor (LET) could promote cell proliferation and inhibit apoptosis of fibroblasts, leading to burn wound healing [4]. Moreover, lncRNA AC067945.2 inhibits collagen expression and ECM synthesis in skin fibroblasts [5]. LncRNA X-inactive specific transcript (XIST) has been reported to be involved in development and therapeutics of many cancers [6]. More importantly, XIST is suggested to promote denatured dermis repair after thermal injury [7]. However, little is known about how XIST could affect the healing of burn wound.

miRNAs are a class of noncoding RNAs with 18–25 nucleotides, which could lead to mRNAs degradation via directly interacting with their 3′ untranslated region [8]. Furthermore, previous work suggested that miRNAs in skin are associated with skin development and wound healing [9]. The family of miR-29, including miR-29a, miR-29b and miR-29c, has been reported to play important roles in ECM synthesis [7, 10, 11]. In addition, miR-29b-3p, a mature miRNA of miR-29b, is associated with collagen synthesis in fibroblasts [12]. Collagen 1 alpha 1 (COL1A1) is suggested to participate in the progression of fibrotic diseases and wound healing [13, 14]. Based on the putative binding sites of miR-29b-3p and XIST or COL1A1, predicted by bioinformatics analysis, we hypothesized that miR-29b-3p and COL1A1 might be involved in XIST-mediated wound healing. In the present study, we investigated the effect of XIST on ECM synthesis, proliferation and migration in thermal injured-HSF cells. Moreover, we explored the potential ceRNA network of XIST/miR-29b-3p/COL1A1.

## Materials and methods

### Patients and clinical samples

A total 25 patients with deep partial thickness burn (n = 25) were recruited from Tongji Hospital Affiliated to Tongji Medical College, Huazhong University of Science and Technology in this research. The samples were harvested from patients during tangential excision of eschar at the 5th day after thermal injure. The normal skin tissues (n = 25) were remnant donor skin from trunk. Approvals of this study were obtained by the Ethics Committee of Tongji Hospital Affiliated to Tongji Medical College, Huazhong University of Science and Technology. All participants have signed the informed consent. All samples were stored at − 80 °C for following analyses.

### Cell culture and treatment

Human skin fibroblast (HSF) cells were purchased from Gefanbio (Shanghai, China). The cells were cultured with RPMI-1640 medium (Gibco, Carlsbad, CA, USA) with 10% fetal bovine serum (Gibco) in an incubator with 5% CO_2_ atmosphere at 37 °C.

To establish the cellular model of thermal injury, HSF cells were challenged in 52 °C water for 30 s, while cells in control group were incubated in 37 °C water for same time [7]. Subsequently, cells were cultured in the incubator at normal.

For cell transfection, small interfering RNA (siRNA) against XIST (si-XIST), COL1A1 (si-COL1A1), negative control (si-NC), pcDNA targeting XIST overexpression vector (XIST), COL1A1 overexpression vector (COL1A1), pcDNA empty vector, miR-29b-3p mimic (miR-29b-3p), miRNA negative control (miR-NC), miR-29b-3p inhibitor (in-miR-29b-3p) and inhibitor negative control (in-miR-NC) were synthesized by Genepharma (Shanghai, China). Cell transfection was performed in HSF cells using LipoFiter™ Liposomal Transfection Reagent (Hanbio, Shanghai, China) when cells reached 60–70% confluence in 6-well plates. After 24 h of the transfection, cells were harvested for thermal injury or following analyses.

### Quantitative real-time polymerase chain reaction (qRT-PCR)

Total RNA was extracted using Trizol-based methods and the concentration and purity were analyzed through a NanoDrop ND-2000 spectrophotometer (Thermo Fisher, Wilmington, DE, USA). The RNA was reversely transcribed to cDNA using Taqman™ miRNA or mRNA Reverse Transcription Kit (Thermo Fisher). The qRT-PCR was performed with SYBR and special primers as follows: XIST (Forward, 5′-AATGACTGACCACTGCTGGG-3′; Reverse, 5′-GTGTAGGTGGTTCCCCAAGG-3′); COL1A1 (Forward, 5′-CCGTGCCCTGCCAGATC-3′; Reverse, 5′-CAGTTCTTGATTTCGTCGCAGATC-3′); β-actin (Forward, 5′-ATGGGTCAGAAGGATTCCTATGTG-3′; Reverse, 5′-CTTCATGAGGTAGTCAGTCAGGTC-3′); miR-29b-3p (Forward, 5′-TGCGGTAGCACCATTTGAAAT-3′; Reverse, 5′-CCAGTGCAGGGTCCGAGGT-3′); U6 (Forward, 5′-TCCGATCGTGAAGCGTTC-3′; Reverse, 5′-GTGCAGGGTCCGAGGT-3′). The samples were prepared in duplicate. β-actin or U6 was regarded as internal reference for XIST, COL1A1 or miR-29b-3p, respectively. Their relative expression levels were analyzed according to the 2^−ΔΔCt^ method [15].

### Western blot

After washed with PBS, HSF cells were harvested for total protein extraction using RIPA lysis buffer (Yeasen, Shanghai, China). Following the high-speed centrifugation at 4 °C, the protein in supernatant was quantified according to the bicinchoninic acid (BCA) method with a BCA protein quantification kit (Yeasen). Equal amounts (20 μg) of proteins were prepared for SDS-PAGE electrophoresis and then transferred to nitrocellulose membranes (Millipore, Billerica, MA, USA) by transmembrane with western transfer buffer (Beyotime, Shanghai, China). Each sample was prepared in triplicate. The membranes were blocked using QuickBlock™ Blocking Buffer (Beyotime), interacted with primary antibodies against Collagen I (ab34710, Abcam, Cambridge, MA, USA), alpha smooth muscle actin (α-SMA) (ab32575, Abcam), COL1A1 (sc-293182, Santa Cruz Biotechnology, Santa Cruz, CA, USA) or β-actin (ab8227, Abcam) overnight at 4 °C, and incubated with special horseradish peroxidase-labeled secondary antibody (ab6721 or ab6728, Abcam) for 2 h. Protein blot was visualized in the dark using BeyoECL Plus (Beyotime) and films (Carestream Health, Rochester, NY, USA). The relative expression of protein was analyzed using Quantity One software (Bio-Rad, Hercules, CA, USA) with β-actin as an endogenous control.

### Cell proliferation

Cell proliferation was measured using Cell Counting Kit-8 (CCK-8) (Beyotime). Transfected HSF cells were seeded into 96-well plates at a density of 3000 cells per well overnight and every sample was prepared in quadruplicate. At 0, 24, 48 or 72 h after thermal injury, cells were incubated with 10 μl CCK-8 solution for another 3 h. Subsequently, the absorbance at 450 nm was determined using a microplate reader (Bio-Rad).

### Trans-well assay

The 24-well trans-well chambers (Corning, Corning, NY, USA) were used for investigation of migrated ability. Thermal injured cells in serum-free medium were seeded into upper chambers (1 × 10^4^ cells) and the lower chambers were added with 500 μl medium containing fetal bovine serum. After a culture of 12 h at 37 °C, cells migrated to the lower surface were stained with 0.1% crystal violet (Sigma, St. Louis, MO, USA) and counted under an inverted microscope (Olympus, Tokyo, Japan) with three random fields.

### Bioinformatics analysis and luciferase reporter assay

Bioinformatics analysis was used for explore the potential targets of XIST or miR-29b-3p by using DIANA tools and TargetScan. The putative binding sites of miR-29b-3p and XIST or COL1A1 were predicted. For luciferase reporter assay, wild type (WT) or mutant (MUT) luciferase reporter vectors targeting XIST or COL1A1 were established in firefly luciferase-expressing pmirGLO vector (Promega, Madison, WI, USA), named as XIST-WT, XIST-MUT, COL1A1-WT or COL1A1-MUT respectively. HSF cells were co-transfected with miR-29b-3p, miR-NC, in-miR-29b-3p or in-miR-NC and WT or MUT luciferase reporter constructs, along with renilla vector using LipoFiter™ Liposomal Transfection Reagent. At 48 h after post-transfection, luciferase activity was measured using a luciferase reporter assay kit (Promega).

### Statistical analysis

All experiments were repeated three times. Statistical analysis was performed using GraphPad Prism 7 software (GraphPad Inc., La Jolla, CA, USA) with the data expressed as mean ± standard deviation (S.D.). The comparisons between groups were conducted by student’s *t* test or ANOVA followed by Dunnett’s test. The spearman’s correlation analysis was performed to analyze the potential linear relationship of expression level of miR-29b-3p and XIST or COL1A1. The difference was significant when *P *< 0.05.

## Results

### The expressions of XIST, miR-29b-3p and COL1A1 are altered during wound healing

**Fig. 1:**
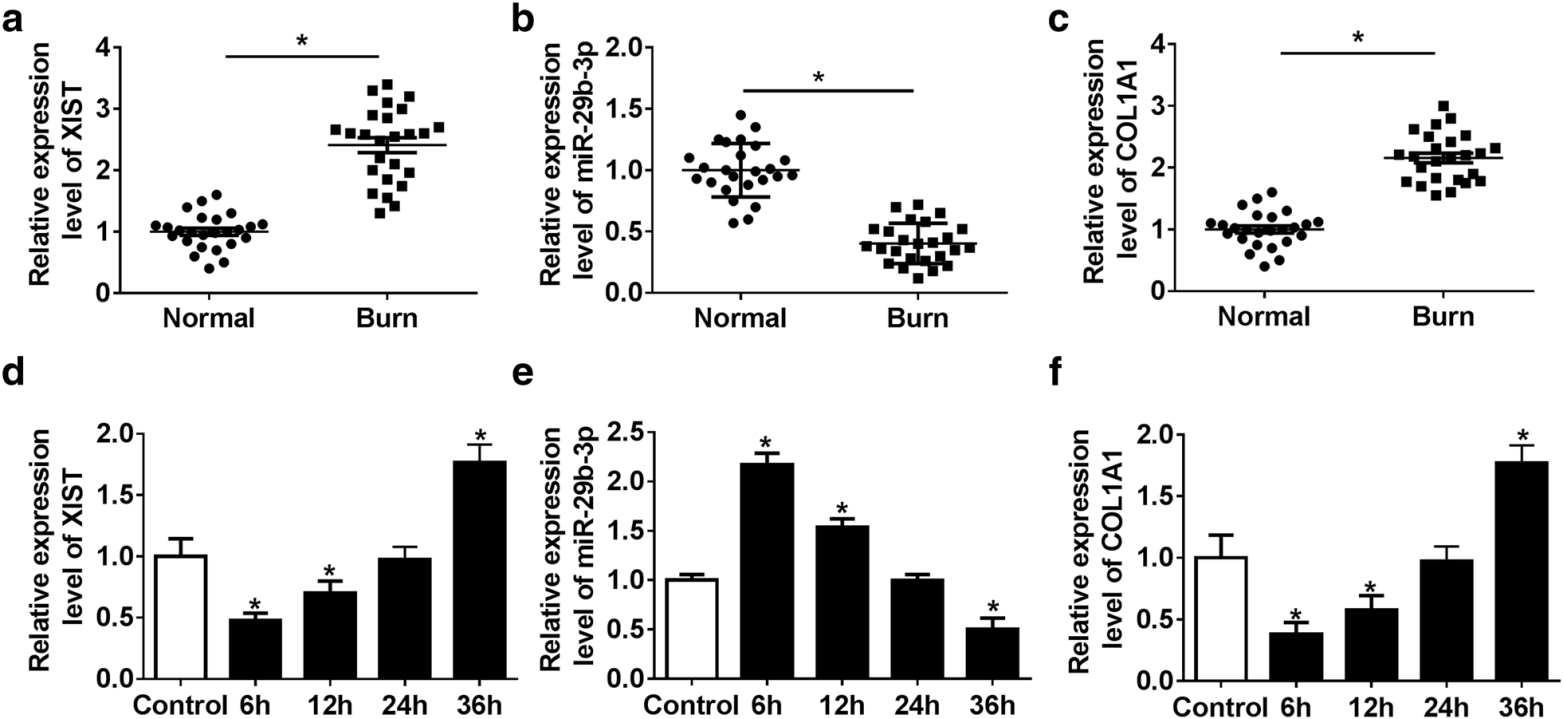
XIST, miR-29b-3p and COL1A1 are aberrantly expressed during wound healing. **a**–**c** The expressions of XIST, miR-29b-3p and COL1A1 were measured in burn wounds and normal samples by qRT-PCR. **d**–**f** The levels of XIST, miR-29b-3p and COL1A1 were detected in HSF cells at 6, 12, 24 and 48 h after thermal injury. *P < 0.05

To explore the potential role of XIST, miR-29b-3p and COL1A1 in wound healing, their expression levels were measured in burn wounds. Compared with those in normal tissues, as shown in Fig. 1a–c, the expressions of XIST and COL1A1 were significantly elevated in burn wounds at day 5 after thermal injure, while miR-29b-3p level was notably reduced. Furthermore, in thermal injured-HSF cells, the levels of XIST and COL1A1 were decreased shortly but gradually enhanced in a time-dependent manner after thermal injury, while miR-29b-3p abundance was gradually reduced at 6, 12, 24 and 48 h after the short increase, when compared to the control group (Fig. 1d–f).

### XIST promotes ECM synthesis, cell proliferation and migration in thermal injured-HSF cells

**Fig. 2:**
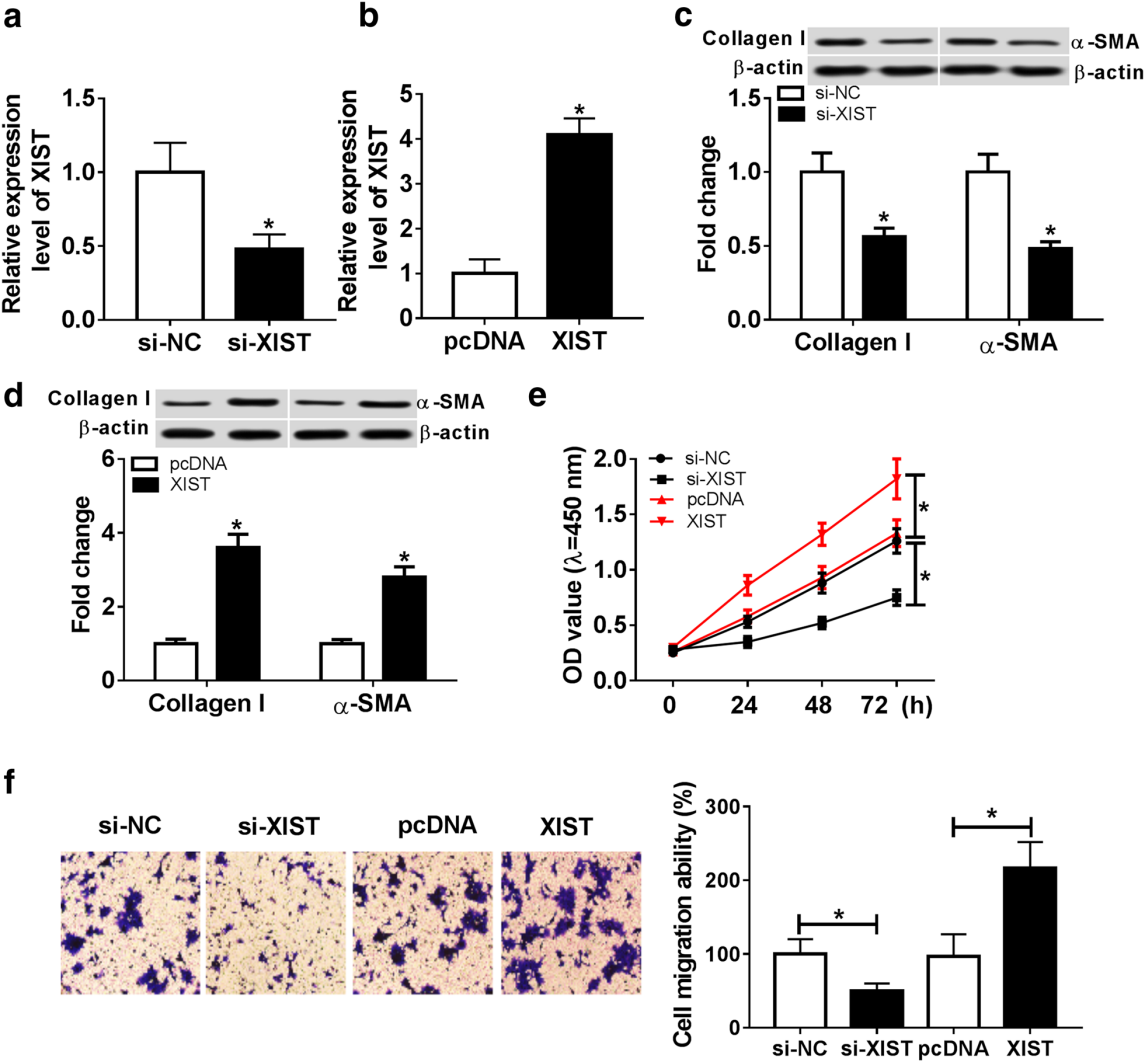
XIST promotes proliferation, migration and ECM synthesis in thermal injured-HSF cells. HSF cells were transfected with si-NC, si-XIST, pcDNA or XIST for 24 h and then suffered from thermal injury. XIST expression level (**a**, **b**), ECM synthesis (**c**, **d**), cell proliferation (**e**) and migration (**f**) were measured in treated cells by qRT-PCR, western blot, CCK-8 or trans-well assays. *P < 0.05

To investigate the biological role of XIST during wound healing, HSF cells were transfected with si-NC, si-XIST, pcDNA or XIST, followed by treatment of thermal injury. The transfection efficacy was validated by reduced level of XIST in HSF cells with transfection of si-XIST and increased abundance in XIST-transfected group (Fig. 2a, b). The ECM synthesis was analyzed with ECM markers detecting by western blot. As displayed in Fig. 2c, d, the expressions of ECM markers, Collagen I and α-SMA protein, were obviously decreased by silence of XIST in thermal injured-HSF cells, while were increased via XIST overexpression. Moreover, XIST overexpression induced cell proliferation in the cells, whereas its knockdown played an opposite role (Fig. 2e). In addition, analysis of trans-well revealed that the ability of cell migration was significantly inhibited by interference of XIST, but promoted via overexpressing XIST (Fig. 2f).

### XIST regulates ECM synthesis, cell proliferation and migration by sponging miR-29b-3p in thermal injured-HSF cells

**Fig. 3:**
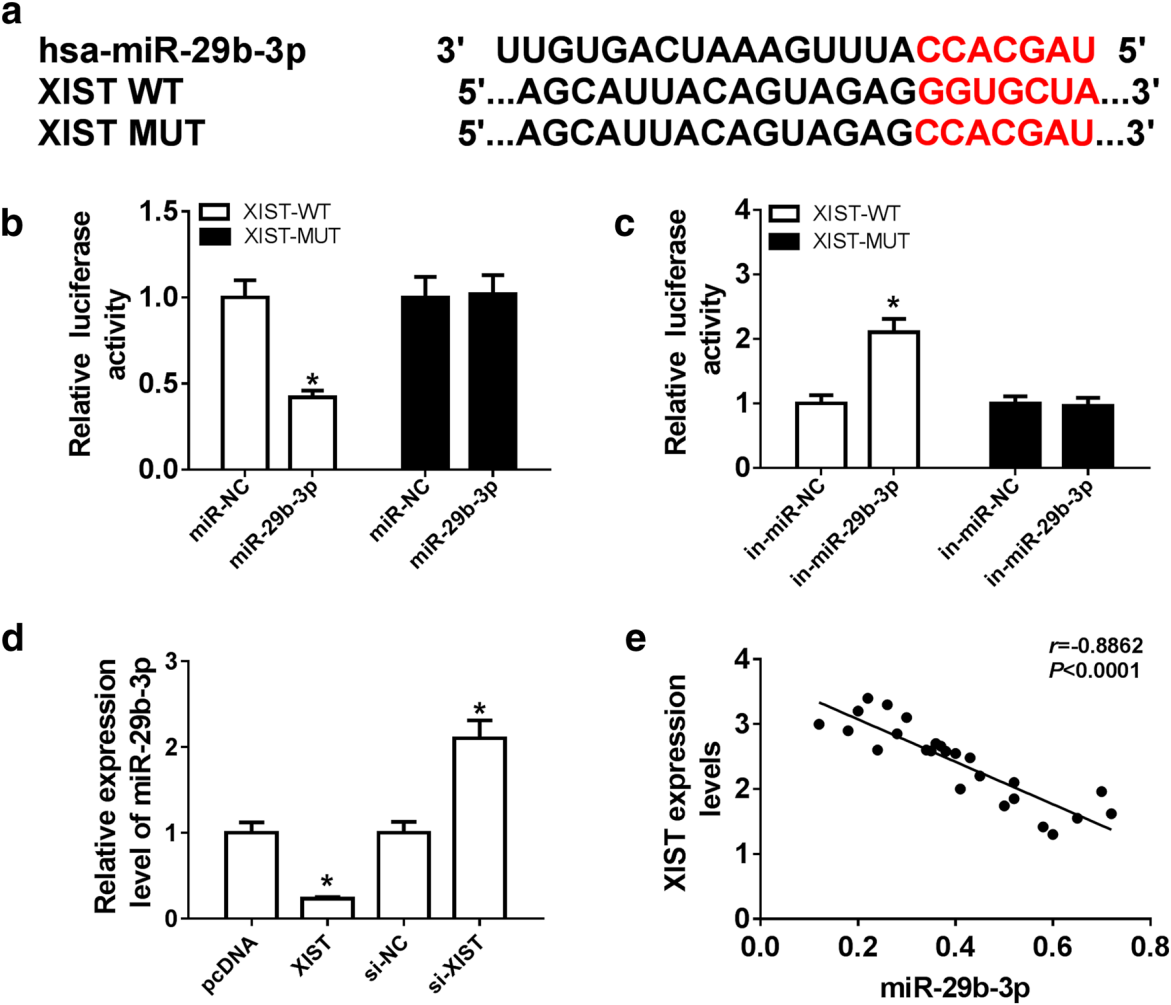
miR-29b-3p is bound to XIST. **a** The potential binding sites of XIST and miR-29b-3p were predicted by DIANA tools. **b**, **c** Luciferase activity was analyzed in HSF cells co-transfected with XIST-WT or XIST-MUT and miR-NC, miR-29b-3p, in-miR-NC or in-miR-29b-3p. **d** The expression of mR-29b-3p was measured in HSF cells transfected with pcDNA, XIST, si-NC or si-XIST. **e** The association between the expression of XIST and miR-29b-3p in burn wounds was analyzed. *P < 0.05

**Fig. 4:**
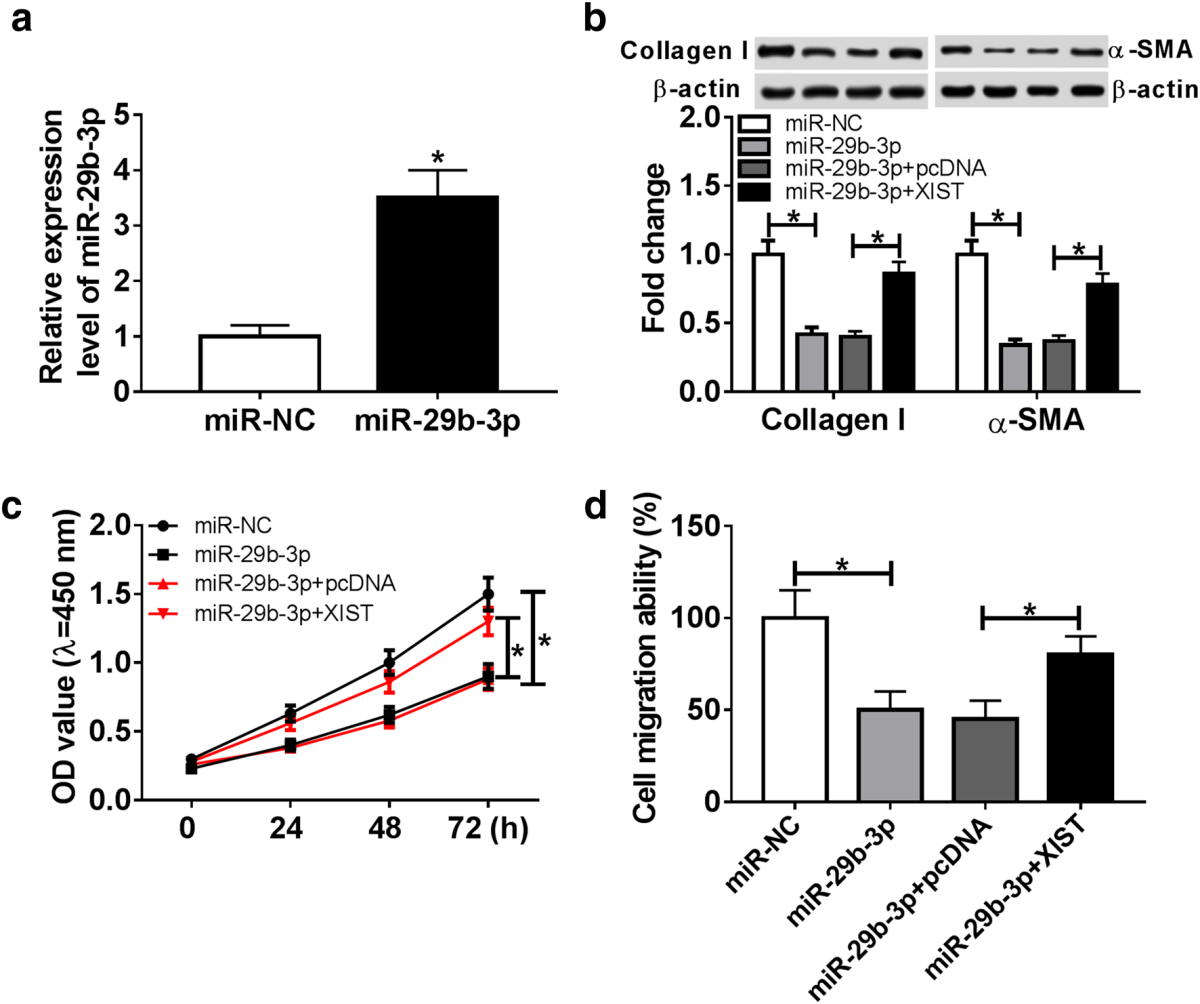
XIST regulates proliferation, migration and ECM synthesis in thermal injured-HSF cells by sponging miR-29b-3p. HSF cells were transfected with miR-NC, miR-29b-3p, miR-29b-3p and pcDNA or XIST for 24 h and then suffered from thermal injury. miR-29b-3p expression level (**a**), ECM synthesis (**b**), cell proliferation (**c**) and migration (**d**) were detected in thermal injured-HSF cells by qRT-PCR, western blot, CCK-8 or trans-well assays. *P < 0.05

To explore the underlying mechanism of XIST during wound healing, the potential miRNAs bound with XIST were searched. Bioinformatics analysis described the putative seeding sites of XIST and miR-29b-3p (Fig. 3a). Moreover, luciferase reporter assay was performed to validate this association with the results which luciferase activity was conspicuously decreased by miR-29b-3p overexpression in HSF cells with transfection of XIST-WT, but increased via down-regulating miR-29b-3p, while it failed the efficacy in response to XIST-MUT group (Fig. 3b, c). Additionally, the effect of XIST on miR-29b-3p expression was evaluated in HSF cells. The results of qRT-PCR assay demonstrated that the abundance of miR-29b-3p was notably decreased by XIST overexpression, but enhanced by XIST silence (Fig. 3d). Meanwhile, the expression of miR-29b-3p in burn wounds was negatively correlated with XIST level (r = − 0.8862, *P *< 0.0001) (Fig. 3e). To explore whether miR-29b-3p-addressed wound healing was mediated by XIST, HSF cells were transfected with miR-NC, miR-29b-3p, miR-29b-3p and pcDNA or XIST before thermal injury. Results showed that expression of miR-29b-3p was effectively elevated in cells after transfection of miR-29b-3p mimic (Fig. 4a). Furthermore, overexpression of miR-29b-3p significantly reduced the expression of Collagen I and α-SMA protein and impeded cell proliferation and migration in thermal injured-HSF cells, which were obviously attenuated by introduction of XIST (Fig. 4b–d).

### miR-29b-3p mediates ECM synthesis, cell proliferation and migration by targeting COL1A1 in thermal injured-HSF cells

**Fig. 5:**
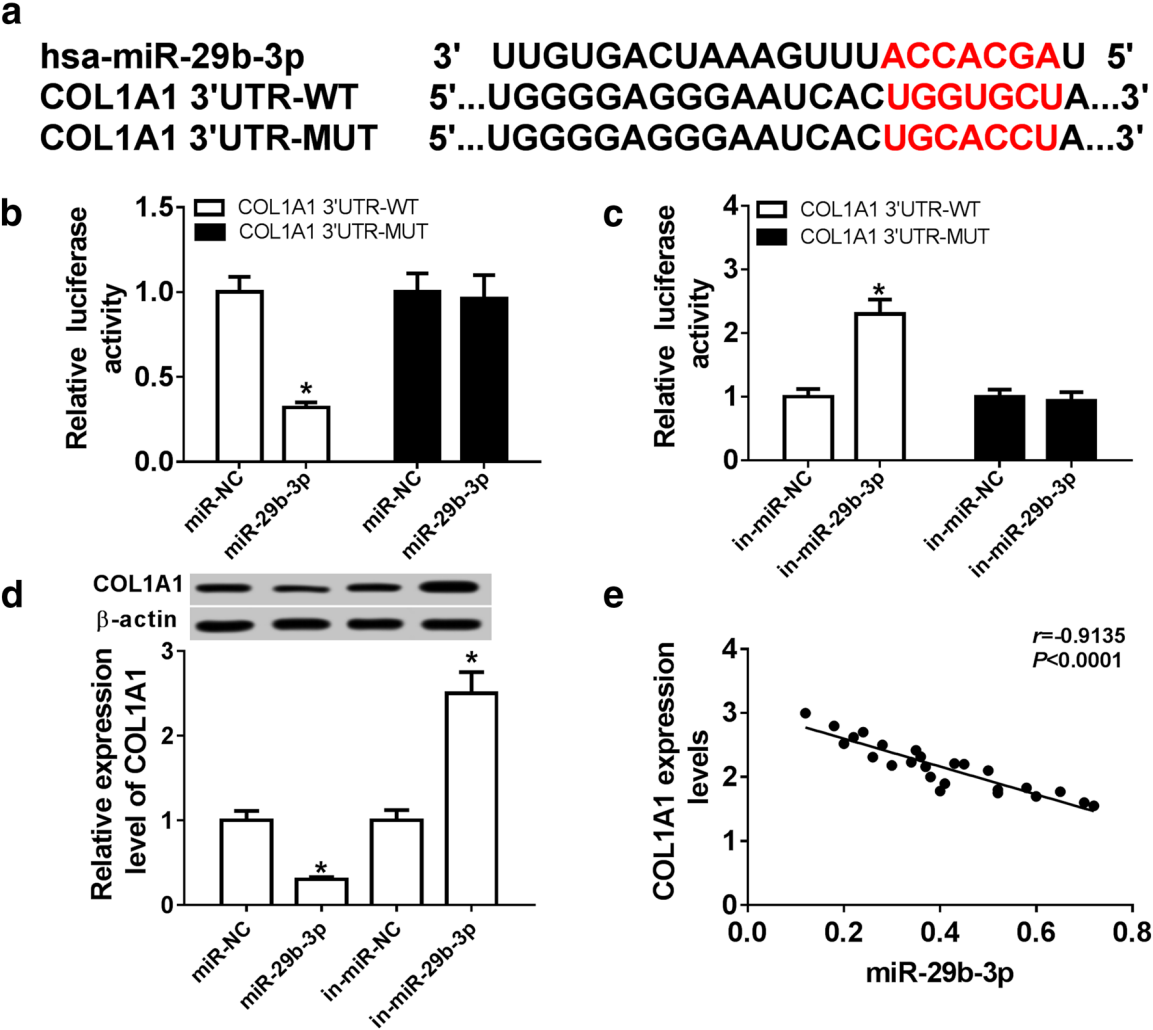
COL1A1 is a target of miR-29b-3p. **a** The putative binding sites of COL1A1 and miR-29b-3p were predicted by TargetScan. **b**, **c** Luciferase activity was analyzed in HSF cells co-transfected with COL1A1-WT or COL1A1-MUT and miR-NC, miR-29b-3p, in-miR-NC or in-miR-29b-3p. **d** The expression of COL1A1 protein was measured in HSF cells transfected with miR-NC, miR-29b-3p, in-miR-NC or in-miR-29b-3p by western blot. **e** The association between the expression of COL1A1 and miR-29b-3p in burn wounds was analyzed. *P < 0.05

**Fig. 6:**
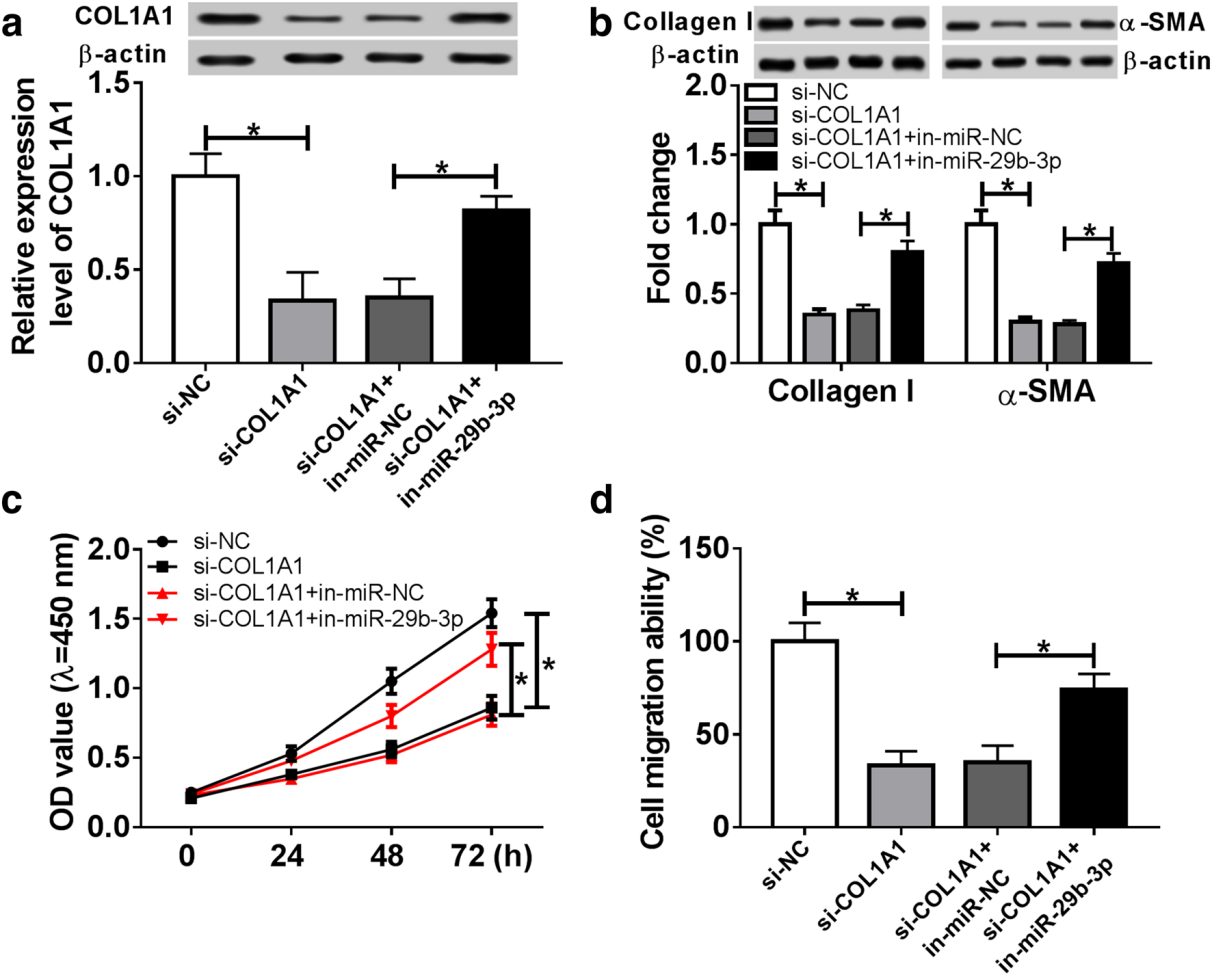
miR-29b-3p abrogation attenuates silence of COL1A1-mediated inhibition of proliferation, migration and ECM synthesis in thermal injured-HSF cells. HSF cells were transfected with si-NC, si-COL1A1, si-COL1A1 and in-miR-NC or in-miR-29b-3p for 24 h and then suffered from thermal injury. COL1A1 protein level (**a**), ECM synthesis (**b**), cell proliferation (**c**) and migration (**d**) were detected in thermal injured-HSF cells by qRT-PCR, western blot, CCK-8 or trans-well assays. *P < 0.05

To further explore the mechanism, the targets of miR-29b-3p were explored by bioinformatics analysis, which showed the binding sites of miR-29b-3p and COL1A1 (Fig. 5a). Furthermore, luciferase reporter assay exhibited that overexpression of miR-29b-3p led to strong loss of luciferase activity in COL1A1-WT group, while its efficacy was lost in COL1A1-MUT group (Fig. 5b). However, miR-29b-3p knockdown caused an opposite effect (Fig. 5c). Subsequently, the impact of miR-29b-3p on COL1A1 protein level was analyzed in HSF cells by miR-29b-3p overexpression or knockdown. The data of western blot displayed that the protein level of COL1A1 was evidently decreased by miR-29b-3p addition and increased by miR-29b-3p deficiency (Fig. 5d). Meanwhile, the mRNA level of COL1A1 in burn wounds was negatively associated with miR-29b-3p abundance (r = − 0.9135, *P *< 0.0001) (Fig. 5e). To explore whether COL1A1 is required for miR-29b-3p-mediated process during wound healing, HSF cells were transfected with si-NC, si-COL1A1, si-COL1A1 and in-miR-NC or in-miR-29b-3p and then treated with thermal injury. After the transfection, the expression level of COL1A1 protein was notably decreased in cells with transfected with si-COL1A1 compared with that in si-NC group, while it was weakened by knockdown of miR-29b-3p (Fig. 6a). In addition, loss-of-function experiment by silencing COL1A1 showed that knockdown of COL1A1 greatly repressed the expressions of Collagen I and α-SMA protein, cell proliferation and migration in thermal injured-HSF cells (Fig. 6b–d). However, these effects were alleviated by down-regulation of miR-29b-3p.

### COL1A1 restoration abolishes the role of XIST knockdown in thermal injured-HSF cells

**Fig. 7:**
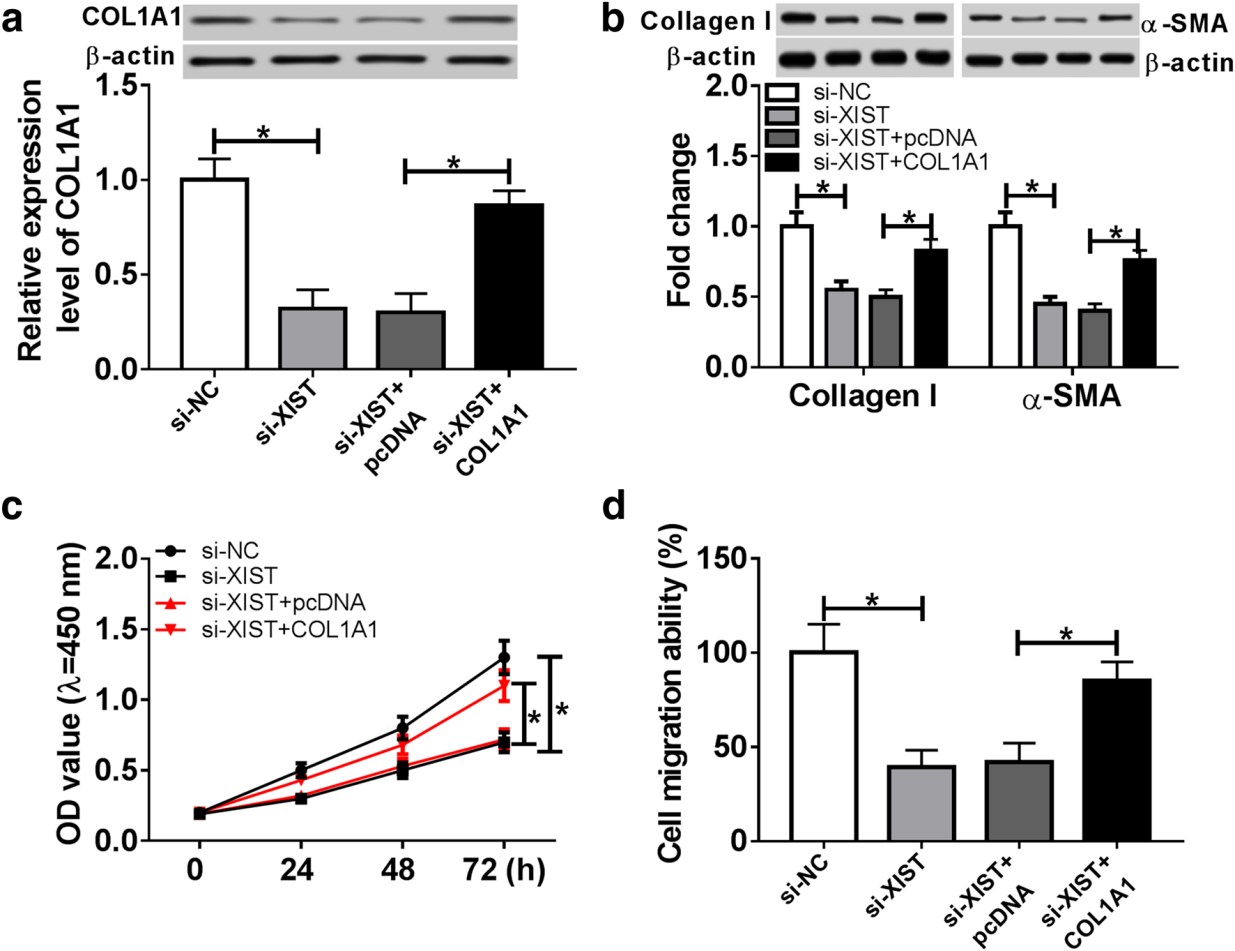
COAL1 reverses knockdown of XIST-mediated inhibition of proliferation, migration and ECM synthesis in thermal injured-HSF cells. HSF cells were transfected with si-NC, si-XIST, si-XIST and pcDNA or COL1A1 for 24 h and then suffered from thermal injury. COL1A1 protein level (**a**), ECM synthesis (**b**), cell proliferation (**c**) and migration (**d**) were detected in thermal injured-HSF cells by qRT-PCR, western blot, CCK-8 or trans-well assays. *P < 0.05

We then investigated whether COL1A1 is involved in the regulatory process of XIST during wound healing, HSF cells transfected with si-NC, si-XIST, si-XIST and pcDNA or COL1A1 were suffered from thermal injury. As a result, the expression of COL1A1 protein was obviously suppressed by silence of XIST, while it was rescued by introduction of COL1A1 overexpression vector (Fig. 7a). Besides, the rescue experiments demonstrated that restoration of COL1A1 significantly abrogated the suppressive effect of XIST interference on ECM synthesis, cell proliferation and migration in thermal injured-HSF cells (Fig. 7b–d).

## Discussion

ECM synthesis and HSF cell function play important roles in wound healing after thermal injury. In the current study, we found that XIST and COL1A1 expressions were enhanced but miR-29b-3p expression was reduced in burn tissues. By establishing a thermal injured model using HSF cells, we found that XIST promoted ECM synthesis and HSF cell proliferation and migration. Besides, this study first explored the interaction among XIST, miR-29b-3p and COL1A1.

Collagen biosynthesis after injury is one of the main markers predicting the healing outcomes. During wound healing, fibroblasts express and secrete α-SMA protein, which exhibits beneficial for constriction. Collagen I and α-SMA are two main markers in ECM synthesis [7]. Furthermore, the activation of HSF cells contributes to restoring tissue integrity. Previous studies demonstrated that XIST knockdown suppressed ECM synthesis and HSF processes [7, 16]. Consistently with these reports, our study also displayed that XIST might accelerate the wound healing after thermal injury by promoting ECM synthesis, HSF proliferation and migration. However, the underlying mechanism remain largely unknown. Cell proliferation is known to be mediated by cell cycle process, apoptosis and autophagy and epithelial–mesenchymal transition production might contribute to cell migration. Whether XIST could influence these cell processes to regulate HSF function is needed to be explored in future. Here we focused on the ceRNA network of XIST in this study.

LncRNAs could block miRNAs expression by serving as sponges or ceRNA in may cancers or diseases [3]. Previous studies have indicated that XIST could function as decoy of many miRNAs, such as miR-140-5p, miR-106b-5p and miR-486-5p [17–19]. This paper first demonstrated the interaction between XIST and miR-29b-3p in HSF cells. Here we found that miR-29b-3p expression was decreased aster thermal injury and its overexpression suppressed ECM synthesis, which is also consistent with former works [20, 21]. However, the expression of miR-29b was not affected in a rat burn model [22]. We hypothesized that miR-29b might not be an important miRNA in rat during wound healing because of the species differences. Moreover, addition of miR-29b-3p suppressed HSF cell proliferation and invasion, which was abrogated by XIST overexpression, suggesting that XIST could mediate wound healing by sponging miR-29b-3p.

The underlying mechanism was further explored, focusing on the target of miR-29b-3p involved in skin remodeling, like collagen synthesis. COL1A1 is associated with synthesis of type I collagen, which plays an important role in skin development during wound healing [23]. Here we confirmed COL1A1 as a target of miR-29b-3p in HSF cells by bioinformatics analysis and luciferase reporter assay, which is also reported by former work [24]. In this study, we found that COL1A1 expression was progressively increased after thermal injury, which is also in agreement with previous study [14]. Loss-of-function experiments displayed that silencing COL1A1 decreased ECM synthesis, HSF proliferation and migration, suggesting that COL1A1 might contribute to wound healing. However, the effect of COL1A1 was regulated by miR-29b-3p and XIST in vitro, indicating that XIST might act as a ceRNA for miR-29b-3p to regulate COL1A1. To further elucidate the role and mechanism of XIST in burn wound healing, an animal model might be helpful in further study. In addition, former work demonstrated that Wnt/β-catenin signaling pathway was implicated in the regulatory role of lncRNA in dermal fibroblasts and skin fibrosis [25]. Hence, whether XIST could address Wnt/β-catenin signaling should be investigated in future.

## Conclusion

Our findings uncovered the potential value of XIST on burn wound healing, revealed by promotion of ECM synthesis, HSF proliferation and migration, possibly by up-regulating miR-29b-3p and down-regulating COL1A1. This study first provided the ceRNA network of XIST/iR-29b-3p/COL1A1, providing a promising avenue for accelerating wound repair after thermal injury.

## Data Availability

The datasets used and/or analyzed during the current study are available from the corresponding author on reasonable request.

## Abbreviations

XIST: X-inactive specific transcript
HSF: human skin fibroblasts
ECM: extracellular matrix
COL1A1: collagen 1 alpha 1
ceRNAs: competing endogenous RNAs
LET: low expression in tumor
siRNA: small interfering RNA
qRT-PCR: quantitative real-time polymerase chain reaction
WT: wild type
MUT: mutant
S.D.: standard deviation
